# Relaxation by Aeroplane

**Published:** 1914-05-30

**Authors:** 


### A DOCTOR'S FIRST FLIGHT.

### Relaxation by Aeroplane.

A description of the sensations of an ignorant passenger on a flying machine may interest some who have not had the fortune to fly, and others who, having been up, may be interested to compare experiences. For a few days, during the absence of a brother medical officer, I was doing duty at the Upavon Flying School, and so the chance to fly came to me. Standing by the sheds on a still, cold, sunny morning, I was watching the machines rising, circling round over the vast expanse of down before me and gliding gracefully back to the aerodrome, when one said to me, " Like to come up with me, Major? " With that there came the first of my sensations, half delight, half funk. Did I want to go up or did I not? I wasn't sure at the moment. Hang it all, it would not do to say No. That will look as if I funked it. The same sort of feeling as comes over you when the next fence looks uncommonly strong and you are doubtful of the size of the ditch on the far side. So I said, "Many thanks, I should very much like it "—Heaven pardons white lies—and walked off with my friend into the sheds to array myself in a pair of goggles, a leathern jacket, and a helmet calculated to save me from breaking my neck if we did not fall very far. A biplane was run out by mechanics and set ready for the flight. " Put your left foot here; your right here. Don't step on the canvas. Sit down there. You can hold on to those uprights." I had successfully climbed to my seat in the machine. My pilot climbed up to the seat in front of me and settled himself. I listened with interest but without understanding whilst he repeated a cabalistic-seeming formula, of which only the word " contact " lingers in my memory.

The great propeller behind me was set in motion by a dexterous twist given it by one of the mechanics. It rapidly increased its velocity. There was a loud roaring sound; dust flew back before the mighty blast. My pilot signalled to let go. We began " to taxi " rapidly but smoothly across the closely cropped turf of the downs. So smoothly did we run that I did not appreciate the moment when we ceased to be of the earth; it was with a sudden shock that I realised that we were in the air and high up in it. Equally suddenly I became aware that something was wrong with the machine. The pilot was sitting quite still except that he turned his head slightly now this way, now that, and made little movements of his arms as he manipulated the various controls. But the machine that ought, I knew, to have been going sixty miles an hour at least, was only just holding its own against a roaring, rushing wind. It kept very steady, just now and again rocking slightly from side to side, and now and again dropping with a scarcely perceptible bump, but it was making scarcely any way, just crawling forward. I could see the world below me slowly passing behind us. I could see the pilot before me was absorbed in his task, and knew he was doing his best for us, but I felt that the catastrophe was inevitable. What a fool I had been to come up! But still the catastrophe was delayed, and I discovered that I was several miles away from the sheds, although I was certain we were scarcely moving. I was filled with amazement. Then I realised that the mighty wind was born of our speed, that the absence of any object on either side of us by which to note progress and the great height we had attained, making objects on the earth appear to move but slowly backwards, had given the sensation of absence of motion. I felt happier; I felt happy, and began to note with interest and pleasure the scenes below me. The world looked clean and bright and pretty. We crossed a hollow of the downs; the sensation was that a vast depth had yawned suddenly below. I could see roads like white ribbons, and sheep-folds in which the sheep standing or lying down looked curiously like the carved sheep in a Noah's Ark. We passed over a farmhouse with a farmstead behind it and ricks in the rickyard. All looked small, pretty, and like a model. The buildings appeared strange and foreign. It was the unfamiliar point of view, no doubt, that gave this sensation. I could see cattle in a field and people standing before the house, and I knew the people were watching our flight because I could see their faces, like dull white plates, turned up to us. I was surprised at the wonderful smoothness of the motion, the absence of an expected sensation of rushing through the air, and at the apparent vastness of the machine; it seemed to spread far round me, a world in itself, far bigger than anything I saw below me.

Near and far the country round was familiar to me; it had been familiar to me from childhood. It was most interesting to sit up there high above it all and to pick out well-known scenes from a new point of view. We were describing a vast circle over Salisbury Plain. I could see the great sheds that house the machines far off on my left hand and recognised Sidbury Hill, Beacon Hill, Martinsell, Savernake Forest, the Pewsey Vale, the Avon Valley, and hundreds of minor features in various directions round me. We continued to circle. We were coming back to the long level before the sheds. The pilot reached back and pulled a wire. The engines ceased to work, all noise hushed, we glided downwards with startling rapidity. The fir trees seemed to rush to meet us. Another movement on the part of the pilot, the engines roared again, and we were rushing at great speed not far above the ground towards the sheds. Again the hand came back, again there was silence. The huge machine glided on, touched lightly, rose slightly, touched again, then rushed along the close-cropped sward, wheeled, and halted, as if by order, before the doors of its shed. I climbed down delighted with my trip, and two days later gladly took a second and last opportunity to renew my experience in the air.

